# Self-Driving Scanning Probe Microscopy: From Acceleration to Discovery and Manipulation

**DOI:** 10.1021/acs.accounts.6c00273

**Published:** 2026-07-14

**Authors:** Ganesh Narasimha, Ralph Bulanadi, Jawad Chowdhury, Rama Vasudevan, Yongtao Liu

**Affiliations:** † Center for Nanophase Materials Sciences, 6146 Oak Ridge National Laboratory, Oak Ridge, Tennessee 37830, United States

## Abstract

Scanning probe microscopy offers a powerful suite of techniques for investigating meso-, micro- and nanoscale phenomena — enabling the exploration of advanced materials, heterostructures, quantum materials, as well as providing insight into surface energetics and reactions. Additional spectroscopic capabilities combined with multimodal imaging allow for spatially resolved characterization of functional material properties. Despite its central role, microscopy is still predominantly conducted through a sequential, operator-driven workflow in which data acquisition, interpretation, and subsequent experimental decisions occur in discrete steps. This approach not only reduces experimental throughput but also limits the effective utilization of increasingly complex multimodal data sets, where critical information may remain unrecognized across real-time data streams.

Recent advances in artificial intelligence (AI) and machine learning (ML) have enabled the concept of self-driving laboratories (SDL) which promise to accelerate scientific research by closing the loop between data acquisition, analysis, and experimental control. Within this broader paradigm, self-driving microscopy (SDM) has emerged as a powerful approach to overcome the limitations of traditional, operator-driven microscopy workflows. In this Account, we discuss the evolution of SDM from automated experimental platforms to fully autonomous systems capable of adaptive, decision-driven operation. We highlight its implementation for real-time data processing and optimization, feature identification, and strategies to efficiently explore across multidimensional parameter spaces. Advanced optimization strategies, including Bayesian optimization and reinforcement learning, allow SDM systems to iteratively refine experimental parameters based on prior observations, prioritizing measurements that maximize information gain. This adaptive approach reduces the total number of experiments required while improving the efficiency of exploration.

Collectively, we showcase how SDM accelerates microscopy through AI/ML integration, enables real-time analysis from high-dimensional data acquisition, while simultaneously guiding the experiments toward discovery. In addition to characterization, the autonomous workflows allow for active material manipulation to create nanoscale artificial structures on demand. We highlight that formalizing experimental strategies into algorithmic workflows enhances integration and scalability across instruments and laboratories. These advances position SDM as a broadly applicable paradigm at the forefront of next-generation experimental science.

## Graphical abstract

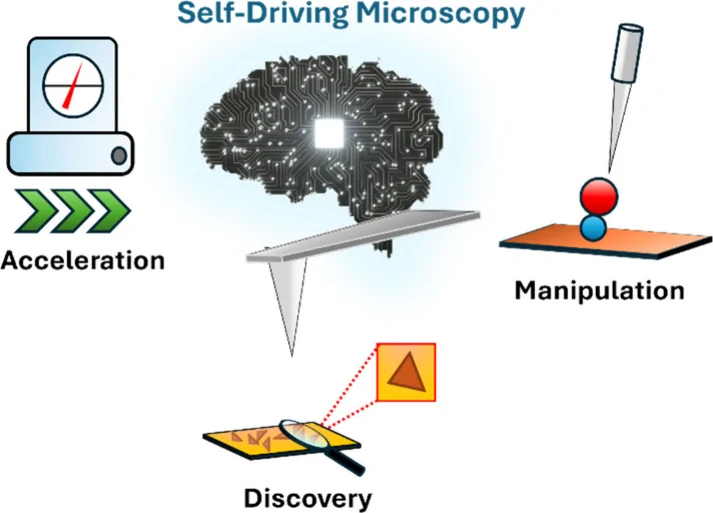

## Key References

-

Liu, Y. ; Roccapriore, K. ; Checa, M. ; Mani Valleti, S. ; Yang, J.-C. ; Jesse, S. ; Vasudevan, R. K. AEcroscoPy: a software-hardware framework empowering microscopy toward automated and autonomous experimentation. Small Methods 2023, 8, 10, 2301740 10.1002/smtd.20230174038639016.^1^ This work represents an application programming interface (API) for microscopy automation and the design principles of API for extending the capabilities of existing microscopy systems.
-

Yang, J. ; Ievlev, A. V. ; Morozovska, A. N. ; Eliseev, E. A. ; Poplawsky, J. D. ; Goodling, D. ; Spurling, R. J. ; Maria, J. P. ; Kalinin, S. V. ; Liu, Y. Coexistence and interplay of two ferroelectric mechanisms in Zn1-xMgxO. Adv. Mater. 2024, 36, 39, 2404925 10.1002/adma.20240492539115333.^2^ This work represents a significant advance in understanding the polarization switching mechanisms in wurtzite ferroelectrics, enabled by automated microscopy.
-

Bulanadi, R. ; Chowdhury, J. ; Funakubo, H. ; Ziatdinov, M. ; Vasudevan, R. ; Biswas, A. ; Liu, Y. Beyond optimization: Exploring novelty discovery in autonomous experiments. ACS Nanoscience Au 2026, 6, 86 41727839 10.1021/acsnanoscienceau.5c00106PMC12921619.^3^ This work shows novelty discovery in autonomous microscopy, in contrast to traditional optimization-driven approaches.
-

Narasimha, G. ; Telychko, M. ; Yang, W. ; Baddorf, A. P. ; Ganesh, P. ; Li, A.-P. ; Vasudevan, R. Automated Construction of Artificial Lattice Structures with Designer Electronic States. ACS Nano 2026, 20, 3393 41362120 10.1021/acsnano.5c12469.^4^ This work demonstrates reinforcement learning driven molecule manipulation and autonomous fabrication of artificial lattices.

## Introduction

Microscopy is a cornerstone for atomic and nanoscale investigation across a diverse set of fields, ranging from electronic and quantum materials to biological systems, and enables correlating atomic and nanoscale structural motifs with local properties.^5–8^ . However, the operational paradigm of microscopy has remained largely unchanged for decades. Traditional microscopy experiments are conducted through manual and operator driven workflows; in this process, data acquisition, data analysis, and decision-making have heavily relied on human experts. These manual processes impose fundamental limitations, as the throughput of experiments is inherently low, making systematic exploration of large parameter spaces practically impossible. Although automated setups in microscopy have always existed, they have been conducted with prescribed logic (e.g., grid spectroscopy or sequential scans), which are simple loop operations and not contingent on results. In addition, the rapidly increasing dimensionality of modern microscopy data, including spatial, spectral, temporal, and multimodal channels,^9,10^ exceeds the capacity of manual analysis. As a result, a significant portion of information remains underutilized when humans are required to make decisions in real-time microscopy experiments, as human operators may overlook important phenomena and hidden patterns due to their inability to analyze and synthesize high-dimensional data rapidly.

Self-driving microscopy (SDM) addresses these challenges by introducing automation and artificial intelligence (AI)/machine learning (ML) for real-time data analysis, feature extraction, and adaptive experimentation.^11–18^ Rapid progress, especially in computer vision methods, triggered a series of developments toward high-throughput image analysis,^19^ defect identification^20,21^ and structure prediction.^22^ While initial developments were restricted to post acquisition inference, the ability to redirect ML informed decisions via automated controls allowed for active learning in real time. This opened possibilities for feature tracking,^23^ inducing structural modifications^24^ as well as scanning tunneling microscopy (STM) tip conditioning.^25^ Further incorporation of physics-informed models within the autonomous loop allowed for models that can be used for hypothesis learning.^26,27^ In these workflows, data is processed immediately upon acquisition, allowing for the extraction of key features and the delivery of rapid scientific insights. When integrated with ML approaches, such as Bayesian optimization (BO) and reinforcement learning (RL), SDM can dynamically adjust measurement conditions based on evolving observations; this reduces the number of measurements required to achieve a given objective by identifying and prioritizing information-rich experiments. In addition, codifying experimental strategies into algorithmic workflows enhances reproducibility and scalability, enabling standardized workflows across different instruments and laboratories.

The role of human experimentalists in designing SDM workflows is paramount. While SDMs are capable of executing workflows without human intervention, the workflow process and the priors are designed by expert knowledge to provide appropriate context for machine inference. This involves (but is not limited to) designing physics-based metrics for model training, determining the relevant context and parameter spaces, formulating quality control mechanisms and performing gated sampling to filter high quality data. Needless to say, interpretation of data is still a heavily expert-driven task, despite significant advances in frontier-model capabilities. These strategies are developed keeping in mind the scaling possibilities of SDMs across experimental modalities.

As such, SDM represents a key step toward accelerated discovery of materials and scientific insights. In this Account, we discuss SDM from the development of automated platforms to the realization of fully autonomous experiments. We explained the application of SDMs in terms of the acceleration of experimentation, discovery, and manipulation of nanoscale phenomena and structures. This highlights opportunities where SDM offers capabilities that transcend traditional experimental approaches.

## Development of Self-Driving Microscopy

To realize SDMs, the first prerequisite is an infrastructure that can seamlessly integrate instrument controls, data acquisition, and analytics. We therefore consider that the development of SDM (Figure 1) as not just a model selection challenge, but necessitates development coordinating both software and hardware.^28^ Traditional microscopy platforms are generally controlled through software provided by vendors; this software is typically designed for interactive operation rather than for streamlining experiments. Vendor-provided APIs enable programmatic control of microscope functions; however, greater flexibility is often required for complex or customized experiments. For example, in the context of PFM experiments the API required to correlate spectral points on a given image is unique to the vendor. Further, specialized experiments such as band excitation are limited to or even unavailable depending on the specific instrument.

**Figure 1:**
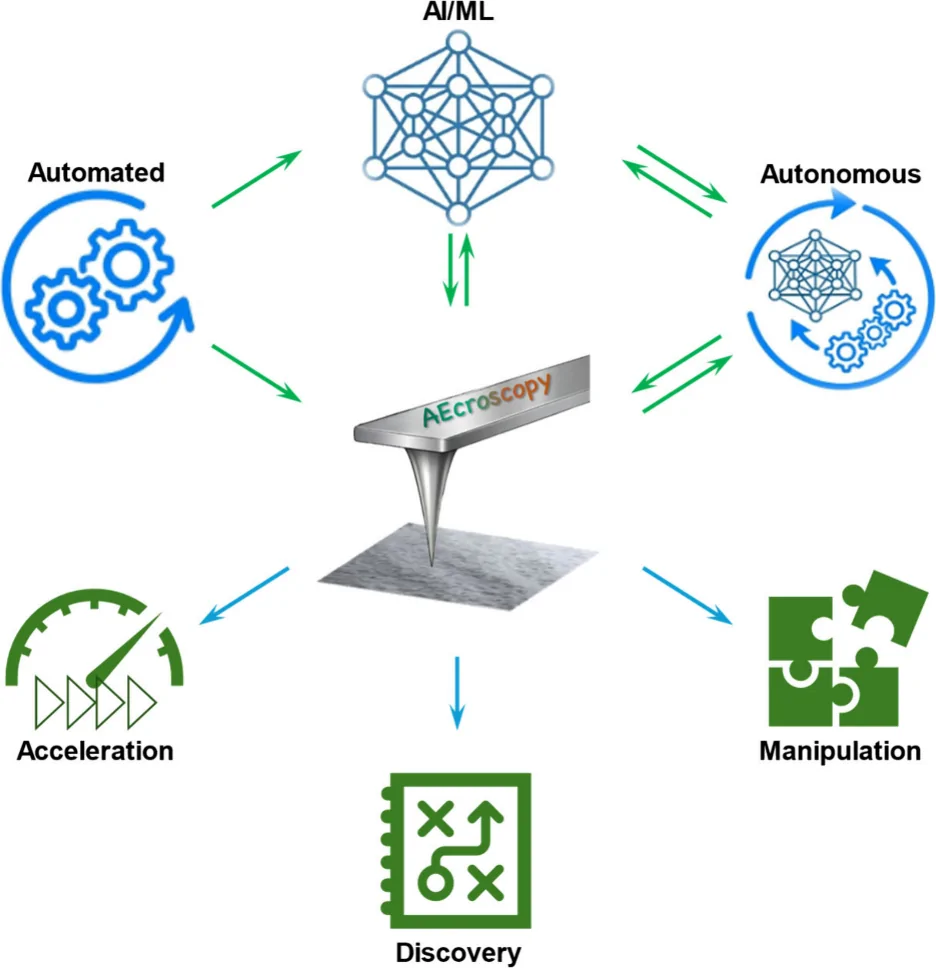
Self-driving scanning probe microscopy (SPM): Integration of automation and AI/ML transforms conventional microscopy into an autonomous platform, accelerating data acquisition, enhancing discovery, and creating functionalities inaccessible through traditional techniques.

To solve this problem, we can integrate additional hardware alongside microscopy systems, coordinated through software orchestration. In this design, the hardware can generate control signals and apply excitation waveforms that are not available within the existing microscope platform, while also enabling high-speed synchronization of measurements. Meanwhile, the software layer manages communication with the microscope. This design enables flexible experiment design while maintaining compatibility with existing microscope platforms, allowing researchers to implement experimental modalities and control schemes that are not accessible through the vendor platform alone.

This parallelly integrable stack led to the development of **AEcroscopy** (short for Automated Experiments in Microscopy),^1^ a software–hardware framework supporting automated and autonomous experimentation in microscopy. In AEcroscopy, a microscopy experiment is no longer a sequence of manual operations, but rather, is expressed programmatically as a workflow composed of ordered operational elements. When executing a workflow, every command (including the function called and the parameters used) issued to the instrument is automatically documented in sequence by logger functions. By describing/documenting experiments in AEcroscopy’s hyper language, the same experimental workflow can be reproduced across different material systems and multiple microscopy platforms without rewriting the procedure. In contrast, manual experimentation typically requires users to repeat the entire sequence of actions for each new sample or instrument, making experiments less scalable and harder to reproduce. In addition, integrating agentic AI with SDM, as exemplified by AEcroscopy,^29^ enables natural language–based operation.^30^ This not only reduces the need for programming expertise to use SDM, but also creates opportunities to connect SDM directly with AI coscientists for real-time hypothesis generation and testing.^31,32^ We envision such agentic approaches, where each element of the workflow (hypothesis generation, simulations, data analysis, etc.) are controlled by agents, to become more practical in upcoming years.

## Automated vs Autonomous

The ability to automate microscopes has enabled both automated and AI/ML-driven autonomous microscopy experimentation. Automated experiments rely on workflows predefined by researchers, where instruments and software execute workflows automatically; these workflows are typically fixed parameters and sequence of operations that improve efficiency, reduce manual intervention, and enhance reproducibility. However, autonomous experiments incorporate AI/ML for real-time data analysis and decision-making, where the results are analyzed in real time and used to dynamically adjust the experiments.^33–36^ This process learns from previous experimental outcomes and adapts the experimental strategy to optimize outcomes or make new discoveries without human intervention. Both automated and autonomous approaches reduce manual labor and improve efficiency, potentially enabling more effective exploration of complex scientific spaces. Beyond saving time and resources, automated and autonomous experiments also enable systematic mapping across spatial, compositional, and external-field variables, allowing generating statistically meaningful data sets and performing experiments that would otherwise be impractical to conduct manually. In this Account, we primarily focus on AI/ML-driven autonomous microscopy based on feedback-controlled mechanisms, i.e., self-driving microscopy. We also present several examples of automated high-throughput microscopy experiments, illustrating how automation alone can reveal subtle changes in materials and phenomena that may otherwise be difficult to detect.

## Acceleration

In microscopy experiments, reliable characterization of a material property strongly depends on characterization parameters. As the number of relevant parameters grows, the search over this space scales exponentially, rapidly increasing search time and cost. ML driven characterization therefore provides an efficient route for navigating high-dimensional parameter spaces via closed-loop tuning of instrument settings for optimizing characterization parameters.^37,38^ For example, BO has been employed to autonomously refine scanning tunneling microscopy (STM) imaging conditions. It does this by learning correlations between imaging parameters (e.g., set point current and tip bias) and image-quality metrics,^38^ through adjusting acquisition policies that balance exploration and exploitation across the parameter space, the system drives rapid convergence to optimal imaging conditions in as little as 20 iterations.

In microscopy, combining imaging with localized spectroscopy is a powerful method to uncover structure–property relationships in materials. Traditionally, these correlative measurements rely on the researcher to determine where measurements should be taken, and which regions are most relevant, but this approach is limited by sparse sampling and potential subjective bias. An alternative approach is grid-based hyperspectral imaging, in which spectroscopy is performed systematically across a predefined spatial grid. Although this approach provides more comprehensive spatial characterization, it often sacrifices efficiency, as many measurements may be redundant for understanding the underlying physics. Additionally, in dense grid hyperspectral measurements, prolonged measurements can introduce secondary effects, such as thermal drift, sample degradation and tip state changes. These time-dependent artifacts can gradually distort spatial alignment and introduce errors in the collected data, further complicating the interpretation of obtained results.

SDM with AI/ML based techniques is well positioned to overcome this limitation, and various ML approaches can be used to guide and accelerate the process of microscopy data acquisition. For example, as shown in Figure 2a–e, we implemented a supervised learning approach to identify structures of interest from image data,^22,27,39–42^ followed by magnifying chosen regions for high-resolution imaging, or spectroscopy characterization for stimuli-induced dynamics at the identified locations. This enables systematic exploration of previously known features, providing a comprehensive data set for more robust insights compared to traditional manual-driven measurements that only acquire data at a few representative locations or conditions.

**Figure 2:**
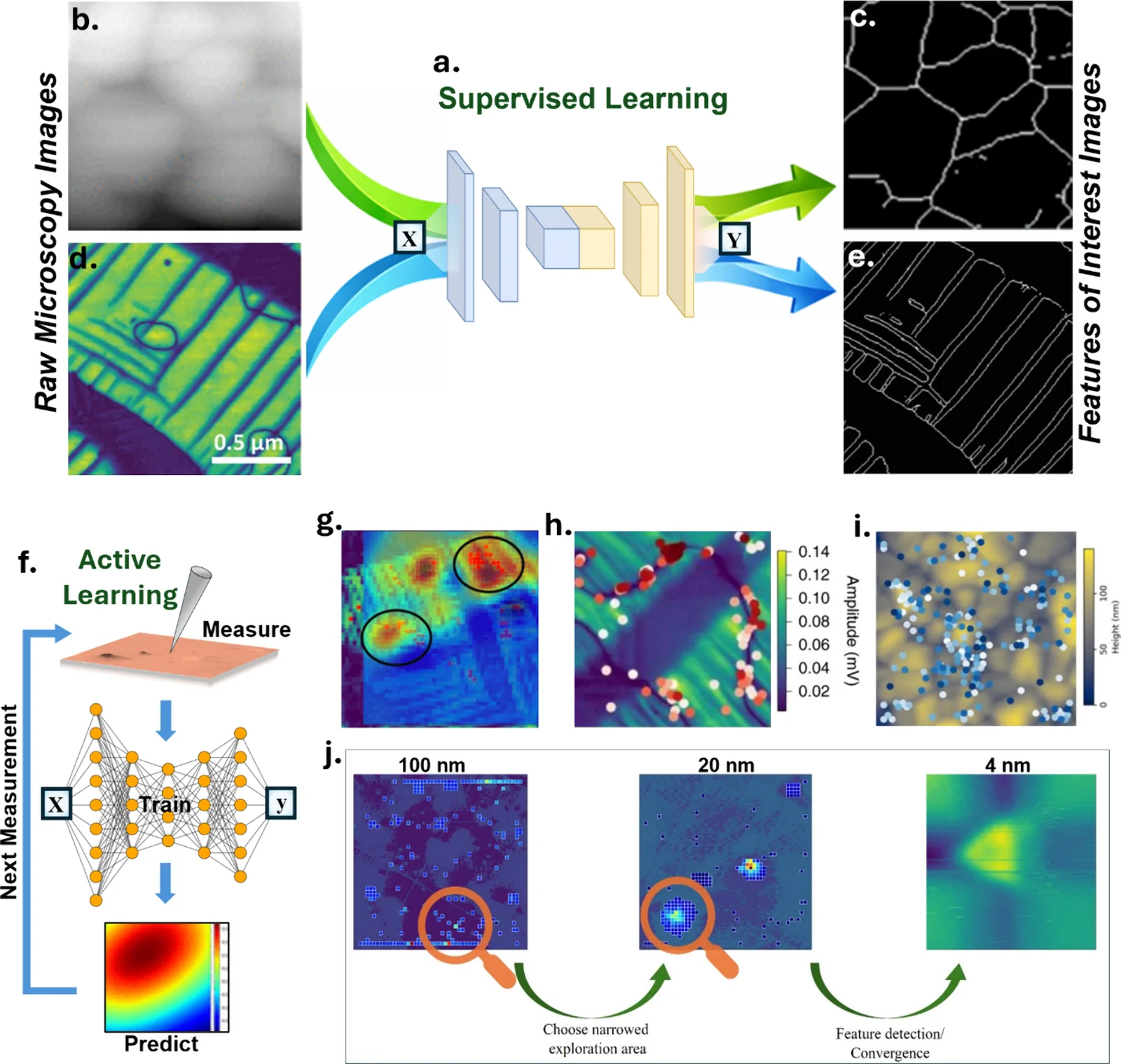
Experimental acceleration enabled by supervised ML and active learning. (a) Illustrates the schematic of supervised learning to extract important features from real-time microscopy images using trained ML models, e.g., (b) → (c) extracts grain boundaries from AFM topography image of halide perovskite thin film (reproduced with permission from ref 39, copyright 2023 American Chemical Society) and (d) → (e) extracts ferroelectric domain walls from PFM amplitude image (reproduced with permission from ref 27, copyright 2022 Wiley-VCH GmbH). (f) Illustration of active learning used in autonomous SPM experiments. Here, the model prediction informs experimental acquisition. (g) Hysteresis loop area from a hyperspectral data overlaid by GP prediction based on a small number of active learning driven measurement; the circled regions indicate agreement at regions that exhibit high loop area (reproduced with permission from ref 49, copyright 2021 American Chemical Society). (h) Points of spectroscopic measurements that were acquired by active learning at the regions that correspond to ferroelectric domain walls (reproduced with permission from ref 44, copyright 2022 Springer Nature). (i) AFM topography of halide perovskite thin film with color-coded IV measurement locations sampled by active learning (reproduced with permission from ref 50, licensed under CC BY 4.0). (j) Shows the multiscale DKL implemented on the STM to automatically locate point defects (reproduced with permission from ref 48, copyright 2025 Springer Nature).

This can be extended to an active learning approach which incorporates subsequent experimental sampling, as illustrated in Figure 2f. The active learning model can be a simple BO which utilizes a Gaussian processes (GP) regressor. For example, Figure 2g shows a comparison of GP-prediction map of regions that have a high characteristic spectral loop area (and therefore indicates strong piezoelectric response) overlaid on experimental acquisitions. While the approximate spatial correlations can be predicted, it does not account for structural features of the sample. This limitation is circumvented in deep kernel learning (DKL) which combines neural net with GP^43,44^ and enables adaptive exploration using an active learning framework. Here, the deep networks efficiently learn the high dimensional correlation within the images while the GP correlates learnt representations with scalar material properties. In ferroelectric materials, it demonstrated capability to identify which structural features can lead to certain piezoresponse-voltage hysteresis characteristics (Figure 2h), such as loop area, coercive voltage, nucleation voltage.^44,45^ When combining piezoresponse force microscopy (PFM) with nonlinearity measurements, it also elucidates relationships between structure and nonlinear characteristics.^46^ In optoelectronic materials like halide perovskites (Figure 2i), DKL driven conductive Atomic Force Microscopy (cAFM) uncovered grain structures with novel current–voltage behavior, as well as insights about grain boundary junctions being less conductive than grain boundary centers, which had been previously missed by conventional cAFM due to the sampling limitations of manual operation.^47^

In STM characterization, real-time DKL for structure-to-electronic-property inference was used to steer autonomous exploration toward regions that optimize a target material property unique to surface termination and point defects.^48^ This was achieved by sparse sampling guided by predicted property maps that required a small fraction of measurements, as little as ∼ 1% in comparison to conventional hyperspectral methods. This paradigm of autonomous characterization can be extended across multiple length scales. In the domain of SPM experimentation, identifying and categorizing diverse surface structures is still largely a manual task where researchers typically interpret features by linking them to material properties using complementary spectroscopic measurements. Furthermore, the process of finding “interesting” structures either relies on exhaustive sample search across multiple length scales or on serendipity. We addressed this bottleneck by demonstrating a multiscale deployment of DKL to address the inverse problem of property-guided structure discovery.^48^ Implemented hierarchically, the approach enables automated identification of the structural origins underlying a measured response. In doing so, the experiment can systematically traverse multiple length scales, discriminate relevant features, and resolve the corresponding atomistic structures. Figure 2j shows an example where a triangular vacancy defect was autonomously located using multiscale deployment of DKL on the STM.

The examples above demonstrate various approaches for learning structure–property relationships, where the properties are represented as scalar descriptors. Supervised learning enables efficient extraction and prediction of features from samples and hyperspectral measurements, while BO supports active learning by constructing a smooth surrogate objective function using GP regression with simultaneous uncertainty quantification. It can be observed that traditional BO is effective for low-dimensional correlations while complex spatial and spectral correlations inherent to imaging data are more effectively captured using DKL.

## Discovery

Microscopy automation also offers opportunities for scientific discovery through both automated high-throughput experiments and AI/ML driven autonomous experiments. In manual operation mode, researchers are often restricted to testing a small number of experiment conditions, whereas automated workflows enable systematic exploration across large parameter spaces, generating statistically rich data sets. This makes it possible to uncover subtle transitions, rare states, and weak correlations that are easily overlooked in sparse and operator-driven measurements.

In recent work, we employed automated PFM to investigate the polarization switching process in wurtzite ferroelectrics.^2,51^ We systematically studied how domain growth varies as a function of DC pulse parameters. In an automated workflow (Figure 3a), hundreds of DC pulse conditions were explored by systematically varying the pulse magnitude and duration, followed by PFM imaging after each pulse. Although domain-writing experiments similar to those can be performed manually,^52,53^ the automated workflow fundamentally changes the scale at which such experiment can be conducted. For example, the automated workflow tested 208 distinct pulse conditions, enabling systematic investigation of targeting phenomena and reducing human bias in parameter selection. This automated workflow generates comprehensive maps of how domains nucleate and grow as a function of the DC pulse parameters, as shown in Figure 3b. It revealed distinct domain regimes in Zn_1–*x*_Mg_*x*_O, including shadow contrast in PFM amplitude image originating from significant polarization switching confined to near surface under low and short pulse conditions, as well as polarization switching thoughout the bulk under high and long pulse conditions.

**Figure 3:**
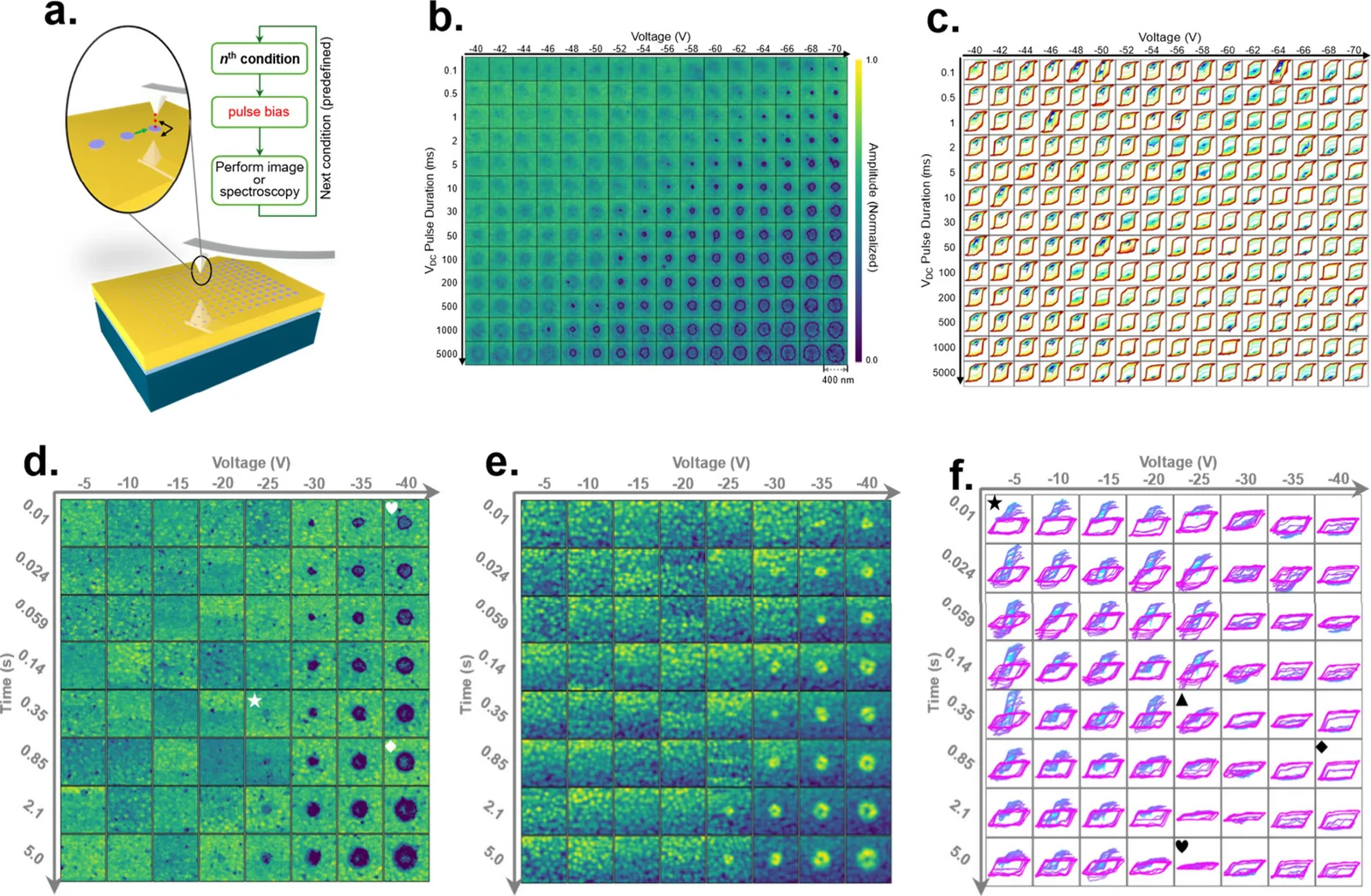
Automated high-throughput experimentation enabled discovery. (a) Experimental workflow: DC pulse-induced switching followed by PFM imaging and spectroscopy to explore domain growth and switching dynamics. (b) High-throughput PFM mapping of 208 pulse conditions showing shadow and normal domains in Zn_1–*x*_Mg_*x*_O (ZMO) film (reproduced with permission from ref 2, copyright 2024 Wiley-VCH GmbH). (c) Evolution of hysteresis loops after varying set pulses in ZMO (reproduced with permission from ref 2, copyright 2024 Wiley-VCH GmbH). (d–e) PFM amplitude and topography results of Al_1–*x*_Sc_*x*_N film across a 64-condition pulse matrix (reproduced with permission from ref 51, copyright 2026 Wiley-VCH GmbH). (f) Evolution of hysteresis loops in Al_1–*x*_Sc_*x*_N film after pulsing (reproduced with permission from ref 51, copyright 2026 Wiley-VCH GmbH).

The significance of automated microscopy extends beyond reducing operator workload and comprehensiveness of experiments, it allows the designed experiments to be adapted to related measurements. For example, the pulse-domain-writing experiment (Figure 3b) can be readily expanded to switching spectroscopy measurements: we used a DC pulse applied immediately before switching spectroscopy measurement, which alters the piezoresponse hysteresis behavior. In these cases, we observed two groups of hysteresis loops (Figure 3c), a group of small loops under low field conditions located within a group of large loops under high field conditions. The small loops observed within the large loops shift downward when the DC pulse increases, correlating strongly with the transition from shadow domains to regular domains in PFM image measurements. This indicates partial switching and global polarization effects, where the small loops originate from partial switching of near surface region and the location of the small loops consistent with global polarization. Based on this observation, we discovered a polarization switching mechanism in wurtzite ferroelectrics, namely, the “fringing ridge” mechanism.

In automated microscopy, once an experimental protocol is developed and encoded into a machine-executable workflow, it can be easily applied across multiple samples, materials systems, and measurement modalities with no or minimal modification. The reusability of automated microscopy workflows was demonstrated by applying them in other material systems such as Al_1–*x*_Sc_*x*_N^2,51^ (Figure 3d–f), where we observed not only multiple step polarization switching but also surface electrochemistry strongly coupled to the switching process. Other automated workflows for physics discovery include automated PFM workflow for composition spread combinatorial libraries of Sm_*x*_Bi_1–*x*_FeO_3_,^54^ enabling systematic study of the ferroelectric–antiferroelectric transition when composition changes.

Automated and high-throughput experimentation provides a pathway toward data-rich materials characterization, unveiling rare states and subtle transitions for physical mechanism discovery, and guiding the design of novel functional materials. It should be noted here that the measurement enabled the acquisition of the data; the interpretation and derivation of the mechanism was still a human discovery. Automated experimentation can displace some of the most repetitive and labor-intensive aspects of SPM experiments, including parameter optimization, repeated measurement execution, and the acquisition of large data sets across multidimensional experimental spaces. As such, researchers can devote greater attention to higher-level scientific activities such as experimental design, mechanistic interpretation, and hypothesis generation and refinement.

When comprehensive measurements are prohibitively slow or expensive, the discovery can be accelerated by implementing AI/ML algorithms that enable autonomous experiments to target regions of high interest. Real-time AI/ML analysis and decision-making for adaptive experimentation enable strategic selection of measurement conditions or regions rather than sampling uniformly and comprehensively. While traditional active learning workflows focus on optimization tasks, such as maximizing a specific physical property, new algorithmic developments have shifted the objective toward discovery, enabling novel phenomena to be uncovered dynamically during the measurement process.

One such strategy is curiosity-driven exploration, in which measurements are guided by the prediction error of a learned structure–property model.^55^ The curiosity-driven workflow (Figure 4a) was introduced to autonomously identify regions where a surrogate model trained on structure to property relationships fails to accurately predict spectroscopic responses. In this framework, an image-to-spectrum (Im2Spec) model learns the mapping between microscopy image patches and the corresponding spectroscopic measurements.^56^ A surrogate error-prediction model then estimates the mismatch between predicted and measured spectra across the sample. Rather than optimizing a predefined physical descriptor such as loop area, the acquisition policy prioritizes locations where the predicted model error is largest – this corresponds to regions where the structure–property relationship remains poorly understood by the Im2Spec model. As the experiment proceeds, the system naturally concentrates measurements on structurally complex or underexplored regions such as domain walls and defect boundaries (Figure 4b), progressively improving the learned model.

**Figure 4:**
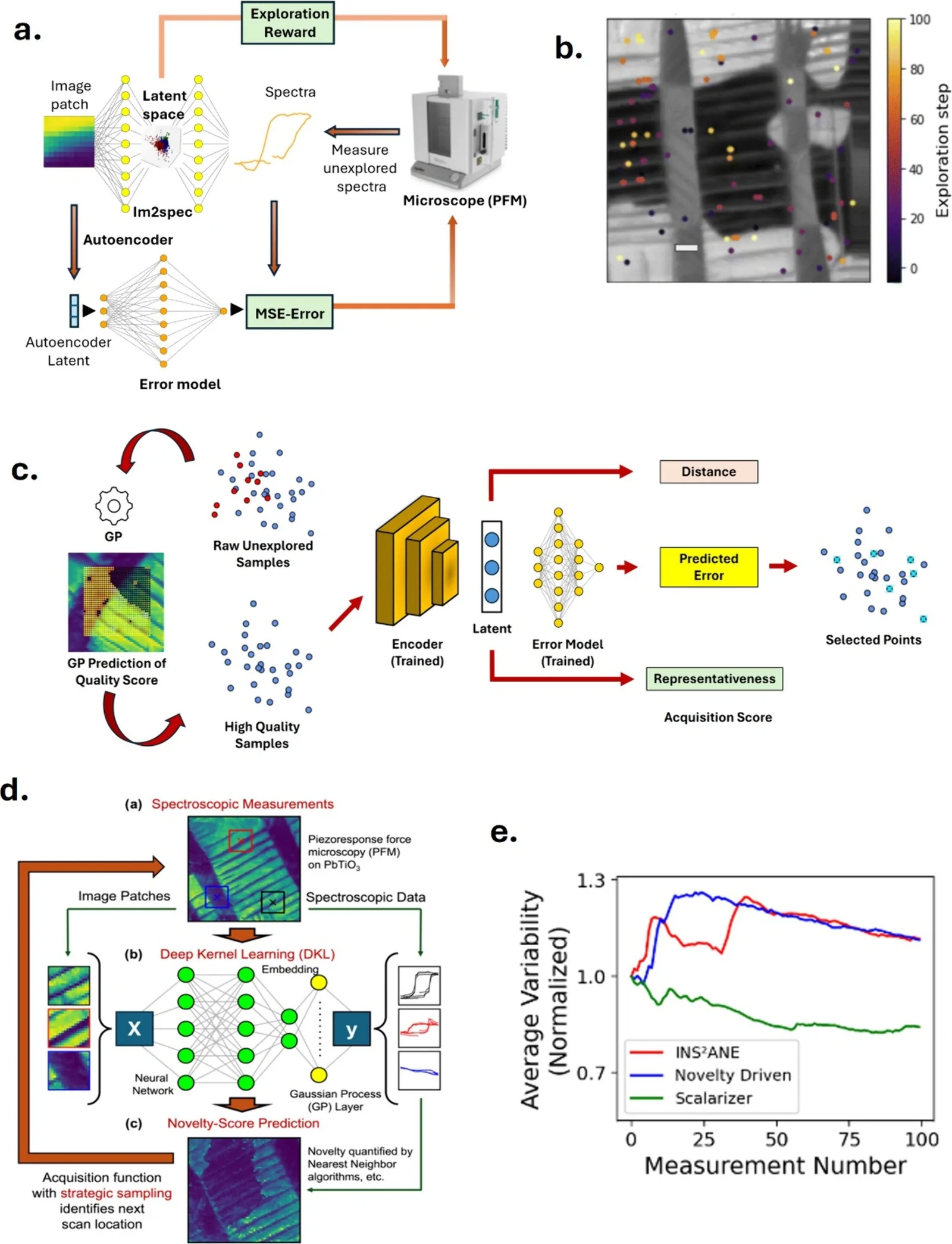
Autonomous discovery in self-driving microscopy. (a) Curiosity-driven active learning guides sampling toward regions where the structure–property relationship is poorly learned. (b) The explored locations in a real autonomous PFM measurements (reproduced with permission from ref 55, copyright 2025 Royal Society of Chemistry). (c) Gated curiosity-driven active learning, where a quality filter excludes low-fidelity measurements and sampling is guided by predicted model error together with heuristics such as distance and representativeness (reproduced with permission from ref 57, licensed under CC BY 4.0). (d) Schematic illustration of novelty driven autonomous experiment, where novelty score computed in the results are combined with strategic sampling to explore unusual or previously unseen behaviors and this is reflected in higher variability of the acquired data as shown in (e) (reproduced with permission from ref 3, copyright 2025 American Chemical Society).

In many practical experiments, however, noisy or low-fidelity measurements can mislead curiosity-based acquisition policies because measurement artifacts may appear as model uncertainty. This spurred the adoption of methods that are quality aware to some extent, ideally governed by underlying physics to impose constraints on the acquisition process.^57^ In such cases, a curiosity-driven strategy can be strengthened by using a gated active-learning framework with quality control (Figure 4c). For example, the implementation for PFM imaging and spectroscopy measurements uses spectral fidelity as a physically meaningful quality metric derived from simple harmonic oscillator fits of switching spectroscopy data. A model then predicts the spatial distribution of measurement quality across the sample, allowing unreliable spectra to be filtered out before selection and restricting exploration to high-quality, reliable regions. The remaining candidates are ranked using a combination of predicted model error and exploration criteria such as distance from existing measurements and representativeness within the data distribution. By integrating curiosity-driven exploration with quality-aware gating, this approach enables robust autonomous learning even in the presence of experimental noise.

Beyond uncertainty-driven exploration, autonomous discovery can also be guided by strategic sampling policies designed to navigate noisy and multimodal experimental landscapes while avoiding premature convergence to local optima. The Strategic Autonomous Non-Smooth Exploration (SANE) framework^58^ addresses the challenge of navigating noisy parameter spaces where conventional BO may become trapped near a local optimum. SANE augments the acquisition process with a strategic exploration policy that probabilistically identifies new regions of interest while balancing exploration and exploitation across the parameter space. A feasibility gate imposed/initialized by domain experts (human-in-the-loop) further constrains the search to experimentally valid regions, enabling the system to explore multiple promising regimes rather than converging inadequately to a single solution. A complementary direction is novelty-driven discovery, where the objective is not a target property but the identification of unusual or previously unseen responses.^3,50,59^ In the Integrated Novelty Score–Strategic Autonomous Non-Smooth Exploration (INS^2^ANE) framework (Figure 4d),^3^ novelty scores are computed in the result data set using anomaly detection algorithms such as nearest-neighbor distance, isolation forests, or one-class support vector machines. These novelty metrics are integrated with the SANE strategic sampling framework to guide experiments toward measurements that are both informative and unusual relative to previously observed data (Figure 4e). By explicitly searching for anomalous responses rather than optimizing a predefined scalar property, this discovery-driven strategy enables autonomous experiments to reveal unexpected physical behaviors that might otherwise remain hidden.

## Manipulation

In addition to material characterization, high-precision feedback-controlled manipulation within the microscope enables deterministic design and stabilization of material states (e.g., domain walls,^60^ artificial lattice,^4,61^ and molecule^62^), effectively turning the instrument into a nanoscale fabrication and control platform. In this mode, the microscope becomes a platform for nanoscale control and functional state engineering (Figure 5a) rather than solely a tool for observation. Here we highlight two distinct examples where we use real-time feedback-based reinforcement learning (RL) methods to realize and encode functional properties using SPM. RL is well suited for dynamic environments, where an agent observes the system state, selects an action, and receives feedback in the form of a reward. In contrast with BO, RL is not myopic – it can operate in the regime of sparse rewards, enabling agents to learn long sequences of actions. This iterative state–action–reward process is used to learn a policy that informs the agent’s action in a given state.

**Figure 5:**
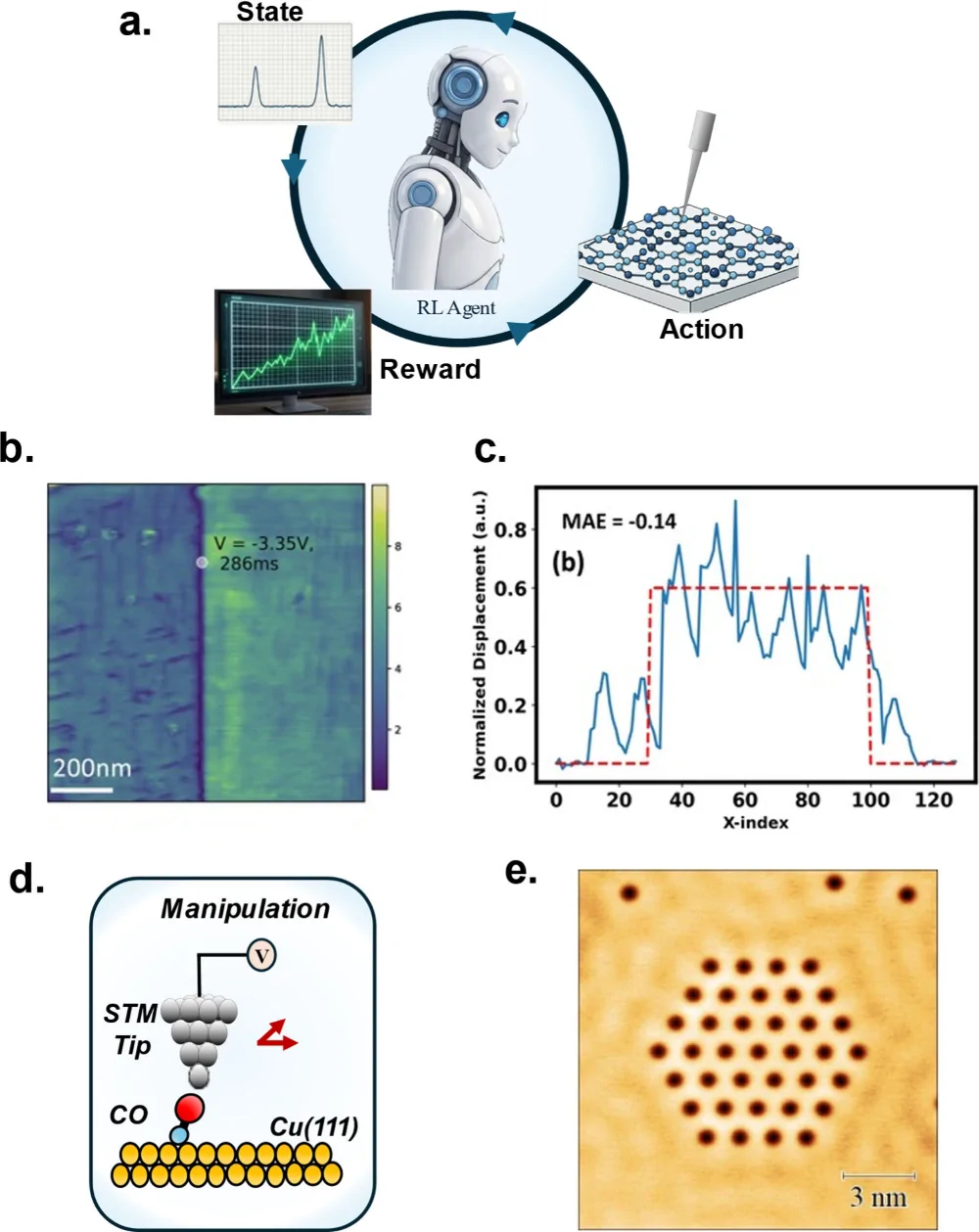
Creation of artificial states using reinforcement learning. (a) Workflow where an RL agent performs an action on the microscopy environment. This involves transition to new states and a corresponding reward; the agent learns to perform optimal action to achieve states that maximize the reward. (b) PFM image, where the tip pulse is applied at the indicated red dot to enable ferroelectric domain wall motion;^60^ data are collected to train an RL model (reproduced with permission from ref 60, copyright 2024 Royal Society of Chemistry). (c) Representative trial with the trained agent: the target wall profile is shown as a red dashed line, and the final wall profile produced by RL agent is shown in blue (reproduced with permission from ref 60, copyright 2024 Royal Society of Chemistry). (d) Schematic illustrating CO molecule manipulation using the STM (reproduced with permission from ref 4, copyright 2025 American Chemical Society). (e) The RL agent constructed artificial graphitic lattice by manipulating CO molecules on Cu(111) (reproduced with permission from ref 4, copyright 2025 American Chemical Society).

The first example is the use of PFM to write prescribed domain-wall geometries in PbTiO_3_ (PTO) thin films. Although PFM is routinely used for imaging and bias-assisted writing, reproducible control of individual wall segments, especially in the presence of pinning and microstructural disorder, remains challenging. We employed autonomous PFM to systematically collect domain-wall responses under DC pulses and used the resulting data to build a physics-informed surrogate model of local wall dynamics under tip-induced electric fields (Figure 5b).^60^ Building on this experimental “environment,” the RL agent learns to optimize tip-bias modification trajectories that reliably produce target domain-wall configurations (Figure 5c). This capability can be combined with engineering strategies to enable ferroelectric topologies as memory storage entities.^63,64^

The second example is the creation of artificial lattices by molecular manipulation using the STM tip.^4^ STM is a well-established platform for positioning adatoms and molecules with atomic precision, enabling engineered lattices that host designer electronic states (Figure 5d).^65^ However, experimentally, the process of manipulation is both time-consuming and sensitive to changes in the tip states. Therefore, in practice, the success of molecule manipulation depends strongly on the manipulation parameters such as current set point and tip bias. In our work,^4,38^ we use RL to determine optimal manipulation parameters to successfully manipulate carbon monoxide (CO) molecules on Cu(111). We first performed offline training, followed by online deployment. Here, the manipulations are carried out across the parameter space, and the data is formulated into a sequence of state, action, reward, and termination, which is then utilized to train the RL agent. This offline stage enables conservative learning from small data sets and supports practical steps such as tuning model hyperparameters, augmenting data, and narrowing the parameter search using expert knowledge. Once trained, the model was deployed on the STM and fine-tuned during experiments. Using this approach, we demonstrated the autonomous construction of an artificial graphene lattice (Figure 5e).

## Conclusion

In this Account, we present the evolution of self-driving microscopy (SDM), from automated experimental platforms to fully autonomous systems capable of adaptive, decision-driven operation. We discuss how SDM accelerates microscopy experimentation through the integration of diverse AI/ML approaches, enables discovery from comprehensive, information-rich data sets and AI-driven exploration of novel phenomena, and facilitates the on-demand creation of nanoscale structures and functionalities. These advances, from faster measurement to autonomous scientific discovery and ultimately on-demand fabrication, positions SDM as a broadly relevant paradigm across materials research and at the forefront of next-generation experimental science. Importantly, these concepts are modality-agnostic, extending beyond scanning probe microscopy to electron microscopy and chemical imaging, further to diverse characterization techniques.

We surveyed a broad range of data-driven methodologies for autonomous microscopy. Supervised learning allows rapid identification of features of interest, while BO and DKL enables structure-property exploration and uncertainty-aware active learning. Beyond scalar property prediction, approaches such as Im2Spec facilitate spectral reconstruction, which can be further integrated with curiosity-driven strategies to promote efficient exploration. RL is particularly well suited for dynamic experimental environments, where sequential decision-making can guide nanoscale material modifications toward desired outcomes. To ensure reliability and reproducibility, these workflows incorporate benchmarking on preacquired data sets, adaptive active learning, automated noise and drift correction, quality-control guardrails, and continuous human oversight.

Looking ahead, agentic AI-driven microscopy^29,30,66,67^ and human-AI collaboration^15,68–71^ represent critical directions for the continued evolution of SDM. Recent developments in AI coscientist systems suggest a future in which intelligent agents can assist not only with experiment execution, but also with knowledge synthesis, hypothesis generation, and experimental planning.^72–74^ Future systems will increasingly operate in a synergistic framework that integrates task-specific AI/ML for execution, agentic AI for reasoning and adaptive refinement, theory-in-the-loop for active parameter inversion and model learning, and human expertise for oversight, technical depth, and high-level guidance. While significant challenges remain before such capabilities can be deployed in microscopy environments, these advances point toward a new paradigm in which autonomous instruments, scientific foundation models, and human researchers operate as an integrated discovery system. Within this paradigm, agentic AI systems enable tightly integrated, end-to-end scientific workflows by connecting SDM with complementary AI models for hypothesis generation, multimodal data fusion, and predictive modeling, forming a closed loop from ideation to experimental validation. Active human involvement in defining experimental goals, constraints, and evaluation criteria will remain essential for accelerating discovery while ensuring scientific relevance.^75^ At the same time, the development of explainable AI frameworks will be critical for enabling real-time interpretation of decision-making processes, allowing researchers to understand, validate, and refine AI-driven actions with confidence.^76^ In parallel, robust quality control frameworks will be necessary to ensure high-quality data inputs for AI/ML models,^57,77–79^ mitigating the impact of measurement artifacts that could otherwise bias training data sets and misguide experimental outcomes.

SDM provides a platform for accelerating materials discovery by enabling rapid characterization and statistical learning of structure–property relationships. Because material properties are often indirectly linked to device-relevant functionalities and may arise from complex, nondeterministic correlations, autonomous workflows can improve reproducibility by capturing batch-level variability and experimental uncertainty. Moreover, reward-driven agents can be designed to identify outliers, sparse feature distributions, and higher-order or nonlocal effects across multiple length scales. It must be noted that while microscopy serves as a powerful characterization tool for probing materials, it does not make new materials. Integrating SDM with other self-driving laboratory platforms, such as autonomous synthesis systems, offers an opportunity to establish distributed, networked experimental ecosystems. In such frameworks, SDM platforms can collaborate across modalities and locations, linking characterization with synthesis to accelerate the discovery and realization of new materials, poising SDM to evolve from a tool for accelerating experiments into a foundational framework for autonomous, collaborative, and end-to-end scientific discovery.
